# Environmental and health risk assessment of polycyclic aromatic hydrocarbons and toxic elements in the red sea using Monte Carlo simulation

**DOI:** 10.1038/s41598-024-71547-4

**Published:** 2025-02-03

**Authors:** F. Alshaima Sayed, Mohamed Hamdy Eid, Ahmed M. El-Sherbeeny, Gouda Ismail Abdel-Gawad, Essam A. Mohamed, Mostafa R. Abukhadra

**Affiliations:** 1 Faculty of Earth Science, Beni-Suef University, Beni-Suef, 62511 Egypt; 2 Shenzhen Key Laboratory of Green, Efficient, and Intelligent Construction of underground metro station, Shenzhen University, Shenzhen , China; 3 Institute of Environmental Management, Faculty of Earth Science, University of Miskolc, Miskolc, 3515 Hungary; 4 Geology Department, Faculty of Science, Beni-Suef University, Beni Suef, Egypt; 5 Industrial Engineering Department, College of Engineering, King Saud University, P.O. Box 800, 11421 Riyadh, Saudi Arabia

**Keywords:** PTE pollution indices, Carcinogenic and non-carcinogenic risk, Monte Carlo simulation, Python code, PAHs, Red Sea, Environmental chemistry, Environmental impact, Risk factors

## Abstract

This research evaluates the environmental and health risks linked to potentially toxic elements (PTEs) and PAHs along the western coast of the Gulf of Suez, Egypt. This study investigated the concentration of 16 PAH compounds in the Suez Gulf, revealing significantly higher levels than the EU (0.20 µg/L) and US (0.030 µg/L) standards. The average total PAH concentration across eight locations was significantly higher, with the Suez area having the highest concentration at 479 µg/L. Pyrene (Pyr) was the dominant PAH with a concentration of 443 µg/L in Suez, while acenaphthylene (Ace) had the lowest concentration at 0.120 µg/L in Northern Zaafarana. Carcinogenic PAHs (CAR) ranged from 8.67 µg/L at Ras Gharib to 29.62 µg/L at Suez, highlighting the urgent need for regulatory measures. Confirmatory ratios pointed to industrial and shipping influences as petrogenic sources. Elevated total organic carbon (TOC) levels in Suez Bay indicated aggravated organic pollution, exacerbated by oil rigs and refineries. The ecological risk assessment highlighted substantial risks, particularly in Suez, necessitating immediate interventions to combat PAH contamination and preserve the environmental balance of the Red Sea. The dominant metals in water samples were arranged in descending order as follows: Pb > Fe > Cr > Cu > Zn > Mn > Cd > Ni. The study evaluated environmental and human health risks using a multifaceted approach, including cluster analysis, principal component analysis, and various indices (HPI, RI, MI, HQ, HI, and CR). Most water samples exhibited high pollution risks, surpassing permissible limits for HPI (> 100) and MI (> 6). Notably, HI oral values indicated significant non-carcinogenic risks for adults and children. While HI values for adults suggested low-risk dermal contact, those for children showed a substantial proportion in the high-risk category. Most water samples displayed CR values exceeding 1 × 10^–4^ for Cd, Cr, and Pb, indicating vulnerability to carcinogenic effects in both age groups. Monte Carlo simulations reinforced these findings, revealing a significant carcinogenic impact on children and adults. The identified clusters, reflective of industrial, petroleum-related, and urban runoff contamination sources, were consistently validated and clarified through PCA, enhancing the reliability of the findings. In light of these results, urgent and comprehensive water treatment measures are imperative to mitigate carcinogenic and non-carcinogenic health risks. These insights provide a foundation for implementing targeted management strategies to effectively address the challenges of heavy metal contamination in the Red Sea.

## Introduction

Environmental pollution from petroleum hydrocarbons, particularly in maritime habitats and inland aquatic breeding environments, poses a significant contemporary challenge. Approximately 5 million tons of crude oil enter the marine environment annually from diverse sources, constituting oil seepage. Various factors contribute to ocean oil spills, including intentional or unintentional ship discharges, tanker collisions, pipeline leaks, well explosions, offshore oil exploration, and onshore refinery leaks. Discharged fuels account for 48% of reported oil pollution, followed by crude oils at 29% and yearly tanker accidents at 5%. Despite a recent decline, the ecological threat from offshore oil spills persists^1^. The consequences of oil spills in estuarine ecosystems attract considerable scientific attention due to intrinsic toxicity to biological components. Approximately five million tons of oil containing harmful compounds enter the marine environment annually through spills worldwide. The process of oil spilling into saltwater involves weathering, including evaporation, natural dispersion, and emulsification. This severely impacts human health and biotic components, such as marine populations, estuaries, coral reefs, and mangroves. Oil contamination poses developmental limitations and socioeconomic risks, endangering marine ecology, the economy, and the human food chain^2^.

Oil components, including water-soluble substances like phenyls, directly harm aquatic life. Fish may absorb hydrocarbons, leading to physiological damage and tainted meat unsafe for human consumption^3^. Insoluble in water, petroleum hydrocarbons create surface films affecting gas exchange and oxygen transport. Polycyclic aromatic hydrocarbons (PAHs), classified as dangerous pollutants, result from oil spills and incomplete fuel combustion. PAHs, known for their toxicity, mutagenicity, and carcinogenicity, can harm aquatic animals^4^. Crude oil, rich in organic molecules, PTEs, and PAHs, poses environmental challenges, impacting marine creatures and ecosystems. The pollutants may enter higher trophic organisms through food chains, affecting coral reefs and mangroves globally. The decline of mangroves is partly attributed to heavy metals and persistent organic compounds acting as natural traps for pollutants^5,6^.

The global concern regarding the presence of heavy metals in aquatic habitats is rooted in the potential harm it poses to human health^7,8^. PTEs are recognized as systemic toxins capable of causing damage to multiple organs, as well as inducing teratogenic and carcinogenic effects^9,9–12^. Prolonged exposure to these metals has been linked to permanent intellectual and developmental impairments, behavioral issues, learning and attention difficulties, auditory challenges, and disruptions of visual abilities^2^. Furthermore, human exposure to PTEs from water bodies occurs through the bioaccumulation of metals in food sources^1,13^. Therefore, individuals can be exposed to high levels of PTEs by consuming plant and aquatic food sources irrigated with polluted waters, even if they do not directly drink water contaminated with PTEs^8,14,15^. These contaminants can originate from natural sources, such as geological erosion, weathering, and precipitation, as well as anthropogenic activities, such as mining, metal processing, industrial wastewater discharge, and applying pesticides and fertilizers^16,17^.

Indices like the heavy metal pollution index (HPI), metal index (MI), and potential ecological risk index (RI) have been utilized to assess the potential ecological risks to the environment^8,18–26^. Additionally, various complementary methods have enhanced analysis efficiency, including multivariate statistical analyses to identify heavy metal sources^8,9,27,28^. The use of the Monte Carlo method in assessing potential risks to human health has proven effective in addressing the challenge of obtaining precise results regarding parameter uncertainty. In recent years, Monte Carlo simulation has been popular in evaluating health hazards related to toxic elements in surface water, groundwater, and soil^8,19,29^.

Responding to water scarcity in Egypt, the increased desalination of Red Sea saltwater serves as an additional drinking water source alongside the Nile River. However, escalating concentrations of PTEs and polycyclic aromatic hydrocarbons (PAHs) in the coastal waters result from diverse pollution sources. Challenges arise in desalination techniques to effectively treat water with high contaminant levels, raising concerns about incomplete removal of heavy metals and pollutants. The local population in the study area directly faces Red Sea water exposure through dermal and oral contact. This emphasizes the urgency of assessing environmental and human health risks associated with pre-desalination heavy metal levels in Red Sea saltwater. Such insights are crucial for implementing effective water treatment strategies, ensuring public health safety, and promoting sustainable water practices in the region^30–32^.

The aims of this study were (1) to evaluate the accumulation and ecological risk of heavy metals, total organic carbon, and polycyclic aromatic hydrocarbons in collected samples from scattered points along the western shore of the Red Sea (2) to carry out a thorough analysis of the potential health risks posed by PTEs (non-carcinogenic and carcinogenic) through oral and dermal exposure for adult and children in the coastal water of the red sea in Egypt; (3) to identify potential sources of the heavy metals by employing cluster analysis and principal component analysis, (4) applying Monte Carlo method to decrease uncertainty and give more realistic approach insight of human health risks from heavy metals that should be considered during desalination of salt water to be utilized safely for drinking and domestic purposes.

## Material and methods

### Site description

The Gulf of Suez (GOS), a semi-enclosed shallow basin stretching 300 km from Port Suez to Shadwan Island, is linked to the northern Red Sea by the Straits of Gubal and the Mediterranean Sea by the Suez Canal. It represents the Red Sea's left arm, which separates Egypt's Sinai Peninsula from Saudi Arabia's Arabian Desert. The Gulf's surface area is around 10,510 km2, with sea depths varying from 16 m south of Suez Bay to 50–85 m near the open Gulf^2,17^. Egypt's main supply of crude oil is the GOS. The GOS oil production, comprising 26 offshore fields, 136 production platforms, and 570 oil wells connected by a submerged pipeline network spanning 450 nautical miles, accounts for 85% of Egypt's crude oil output. The daily canal traffic grew by more than 56 boats, from 18,830 in 2020 to 20,694 in 2021, when the new Suez Canal project was finished in 2015. Thus, the GOS's influence on the Egyptian economy increased considerably^2^.

As a result, there have already been several oil spills in the Gulf that have significantly impacted the marine and coastal ecosystems. There have previously been significant oil leak incidents in the Red Sea/Gulf of Suez (GOS) and the Suez Canal, the worst of which was in 1982. During the loading of a ship in Ras Shukheir, tens of thousands of tons of crude oil were spilled into the sea within 4–6 h. Based on research, the spread of oil pollution was 250 km south and 200 km north of the oil spill. A tanker grounded in the Suez Canal in 2004 spilled 9000 tons of crude oil, and the same thing happened again in 2006 when a tanker sank on the canal bank, spilling 5000 tons of crude oil. The most significant oil leak in Egyptian history, the Gabal El Zeit disaster, happened in June 2010, damaging nearly 160 km of North Red Sea shoreline. Furthermore, it has been stated that the traffic crossroads offshore the Sumed oil pipeline terminal near Ain Sukhna port are the places with the highest risk of tanker accidents^2,33^.

Anthropogenic oil spills from oil rigs and ships have badly impacted the intertidal zone in the middle and southern Gulf of Suez. Many rocky coasts are covered with oil pavements, and in certain coastal regions, oil is discovered buried below a thin veneer of wind-blown sand. The expected distribution of oil contamination sources along the research region is diverse. To explain, although Suez City is prone to oil pollution due to navigation, industrial, manufacturing, and other human activities, Ain Sukhna is rarely contaminated as the area is dominated by tourism and fishing activities^33,34^.

Additionally, the coastal Red Sea and Gulf of Suez region is distinguished by vast areas of mangrove swamps and has a diversified and almost unique coral reef community that serves as a habitat for various marine life, including fish species with significant economic value. Potential toxic elements, petroleum compounds, and herbicides pose the greatest dangers to the Red Sea's coral reefs and mangrove ecosystems. Pollutants can potentially harm coral species, the accompanying creatures, and biodiversity in general. Therefore, to understand the potential dangers, it is crucial to measure the concentration of hydrocarbons, particularly PAHs, and heavy metals in aquatic habitats related to coral reefs and mangroves^35^.

### Sampling and analysis

**Fig. 1:**
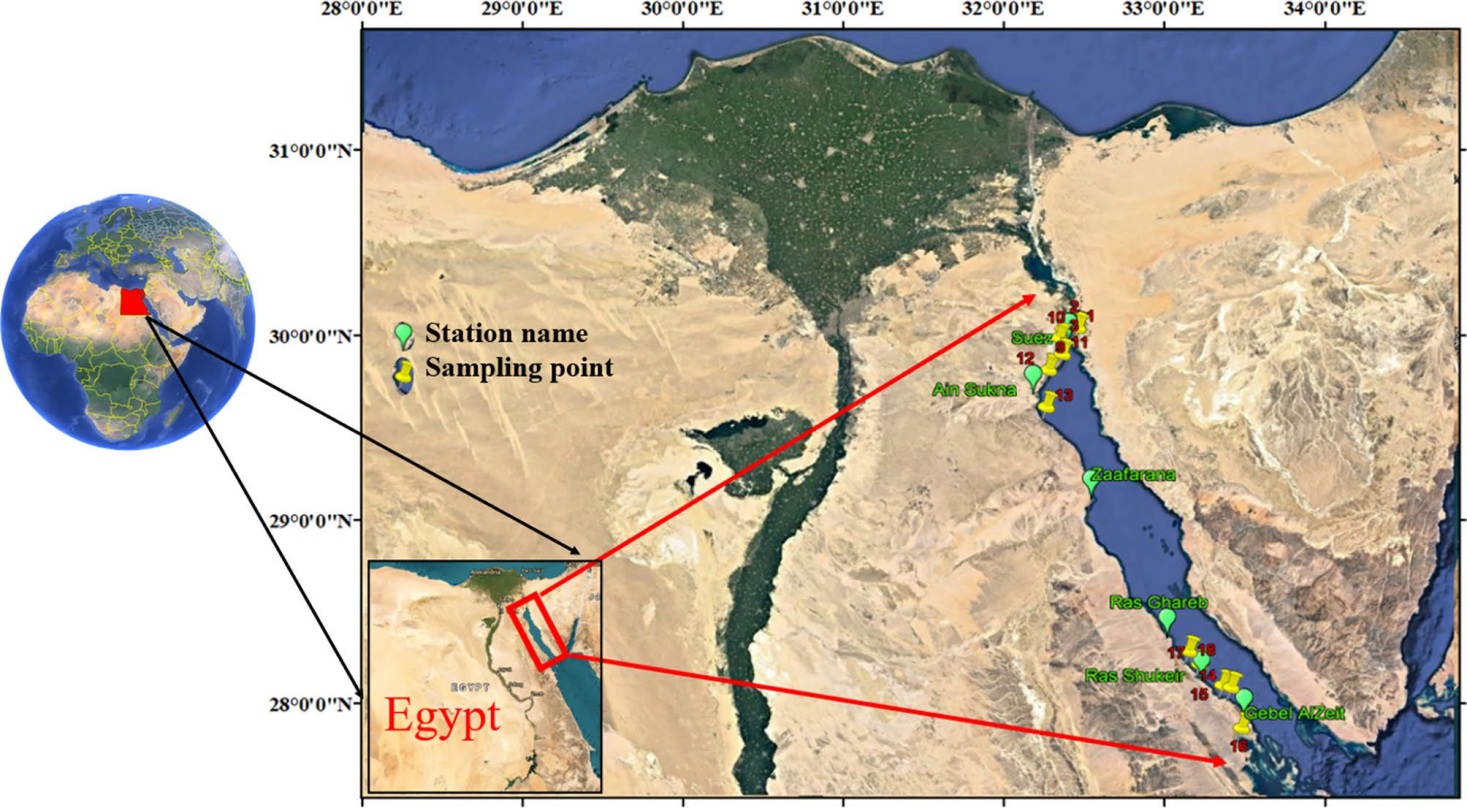
Location map for the area of study.

Water samples were gathered from 18 spots along the coastline of the Gulf of Suez (GOS), as shown in Fig. 1. The map for sampling was made using ArcGIS Pro 2.8.8 software. Sampling of surface water was done on March 17, 2023. In total, 30 samples were collected, with PAHs found in 10 samples and heavy metals analyzed in 18 samples to ensure area coverage at intervals. To guarantee the reliability and accuracy of data, duplicate water samples were taken at each sampling location. Surface water samples were drawn from a depth of half a meter and preserved in four-liter brown glass bottles to shield them from exposure that could degrade certain compounds. Upon arrival at the lab, all samples underwent filtration using filters with a size of 0.45 μm, under vacuum conditions, to separate suspended particles and obtain dissolved samples.

The concentrations of metals were assessed utilizing a coupled plasma optical emission spectrometer (ICP OES), specifically the iCAP 6500 Duo (Thermo Fisher Scientific, England) equipped with a charged injection device (CID) detector. The instrument was calibrated using a certified solution containing elements at a concentration of 1000 mg/L (Merck, Germany). Calibration was done at a concentration of 0.05 parts per million in line with the instructions provided by the manufacturer and following the USEPA Method 6010b (1996). Each sample was tested twice to verify the accuracy of the findings. Additionally, blank samples were examined to determine any background pollution. The detection limits for the heavy metals were as follows: Cadmium (Cd): 0.0001 mg/L, Chromium (Cr VI): 0.001 mg/L, Copper (Cu): 0.001 mg/L, Iron (Fe): 0.001 mg/L, Manganese (Mn): 0.0005 mg/L, Nickel (Ni): 0.001 mg/L, Lead (Pb): 0.001 mg/L, Zinc (Zn): 0.0005 mg/L. The external standards showed recovery rates between 74 and 123% for all elements tested, demonstrating the method's ability to measure metal concentrations accurately. Duplicate samples from the location had standard deviations (RSDs) below 10% for all metals, indicating high precision.

To analyze PAHs, 3 g of each water sample underwent extraction using 5 mL of acetonitrile and sonication for 15 min. Ensuring reliability extractions were performed in triplicate. Following extraction, the samples underwent centrifugation at 3000 rpm for five minutes. The resulting supernatant was filtered through PTFE 0.45 μm filters before undergoing high-performance liquid chromatography (HPLC) analysis. The HPLC system employed was an Agilent 1260 series equipped with a ZORBAX Eclipse PAH column (4.6 mm × 150 mm i.d. five μm). The mobile phase comprised water (A) and acetonitrile (B) with a flow rate set at 2.0 ml/min. The gradient programming was as follows: starting at time point zero with 40% B; from zero to 0.66 min maintaining at 40% B; from 0.66 to 20.0 min increasing from 40 to 100% B; holding at 100% B from minute20 to minute25; decreasing back to 40% B from minute25 to minute27; finally reaching again40% B from minute27 to minute30.Detection occurred at 220 nm utilizing an array detector with an injection volume of 5 μL. The detection limits for various PAHs were: Naphthalene (Naph): 0.05 μg/L, Acenaphthylene (Acthy): 0.10 μg/L, Acenaphthene (Ace): 0.10 μg/L, Fluorene (Fl): 0.05 μg/L, Phenanthrene (Phe): 0.05 μg/L, Anthracene (Ant): 0.05 μg/L, Fluoranthene (Flu): 0.05 μg/L, Pyrene (Pyr): 0.05 μg/L, Benzo (a) anthracene (BaA): 0.05 μg/L, Chrysene (Chry): 0.05 μg/L, Benzo (b) fluoranthene (BbF): 0.10 μg/L, Benzo (k) fluoranthene (BkF): 0.10 μg/L, Benzo (a) pyrene (BaP): 0.05 μg/L, Dibenzo (a,h) anthracene (DBA): 0.10 μg/L, Benzo (g,h, i) perylene (BghiP): 0.10 μg/L, Indeno (1,2,3-cd) pyrene (InP): 0.10 μg/L. Stringent quality control measures were implemented, which used standards to adjust for losses during extraction and preparation. Accelerated solvent extraction was utilized for PAHs with the inclusion of six standards to guarantee compensation. The recovery rates of these standards varied from 76 ± 9% to 118 ± 8%, highlighting the efficiency of the approach. The methods for identifying metals and PAHs in water samples underwent validation to guarantee precise and accurate results. The detection thresholds, recovery rates, and measurement consistency demonstrate the reliability and effectiveness of these techniques for surveillance.

### Environmental risk

The environmental risks in the current study include Polycyclic aromatic hydrocarbon Risk, Ecological risk, and Heavy metals pollution index and metal index.

#### Polycyclic aromatic hydrocarbons risk

The ecological risk of PAHs on aquatic biota was evaluated by risk quotients (RQ). The following Eq. (1) calculated the RQ:

1 $$\text{RQ}=\frac{{\text{C}}_{\text{PAHs}}}{{\text{C}}_{\text{QV}}} .$$

The C_PAHs_ represented the PAH concentrations in the seawater, and C_QV_ represented the quality value of PAHs in the seawater. The negligible concentrations (NCs) and the maximum permissible concentrations (MPCs) of individual PAHs in water represent the quality values, respectively. Hence, the RQ_NCs_ and RQ_MPCs_ were calculated according to Eqs. (2) and (3), respectively^36^:

2 $${\text{RQ}}_{\text{NCs}}=\frac{{\text{C}}_{\text{PAHs}}}{\text{NCs}} ,$$

3 $${\text{RQ}}_{\text{MPCs}}=\frac{{\text{C}}_{\text{PAHs}}}{\text{MPCs}} .$$

#### Ecological risk

The application of the potential ecological risk index (RI) for PTEs (Eq. 4) proves to be a valuable methodology for assessing the potential environmental risks associated with PTEs^37^ In the surface water. Considering factors like concentrations, types, sensitivity, toxicity, and background levels of heavy metals, this approach allows for a comprehensive evaluation of ecological risks. The utilization of this method sheds light on the potential impact of heavy metal contamination on the Red Sea's marine ecosystem and aquatic life. The significance of monitoring and evaluating these risks cannot be overstated, especially in human activities such as desalinating Red Sea water for various purposes. This method offers a practical and insightful tool for understanding the marine environment's overall health and informs decisions to preserve the Red Sea's ecosystem.

4 $$RI=\sum {E}_{r}^{i}={T}_{r}^{i}\times \left\{\frac{{C}_{ave}^{i}}{{C}_{bg}^{i}}\right\},$$

where T_*r*_ means heavy metal toxic response factor, C_*f*_ is the contamination factor; ${C}_{ave}^{i}$ is the concentration of heavy metal; ${C}_{bg}^{i}$ are the background values. *RI* refers to contamination's potential ecological risk.

#### Heavy metals pollution index and metal index

The Heavy Metal Pollution Index (HPI), as explained is considered an effective tool for assessing water contamination levels in the presence of heavy metals (HMs) in water resources^8,38^. This method is valuable for decision-makers, providing a composite measure of the impact of dissolved heavy metals in water. The HPI evaluates water quality suitability for human consumption based on metal contamination, using a weighted arithmetic mean mechanism and assigning weights to each pollution parameter. The HPI is calculated based on the concentrations of eight heavy metals and one metalloid, considering their toxic characteristics. A three-class scale categorizes heavy metal pollution as low, medium, or high. Another metric, the Metal Index (MI), evaluates the overall quality of drinking water by considering the cumulative potential impact of PTEs on human health^8,39^. The MI assumes a linear relationship between the toxicity of HMs and their concentration, with concentrations exceeding allowable limits indicating degraded water quality. The MI, introduced by Tamasai and Cini^40^, is calculated through a comprehensive assessment of the current situation. The following equations were utilized for the calculation of HPI (Eqs. 4 and 5) and MI (Eq. 6)

5 $$CapHPI=\frac{{\sum }_{i=1}^{n}{W}_{i}{Q}_{i}}{\sum_{i=1}^{n}Wi},$$

6 $${Q}_{i}={\sum }_{i=1}^{n}100 \times \frac{{C}_{i}}{{S}_{i}},$$

7 $${M}_{i}={\sum }_{i=1}^{i}\frac{{C}_{ave}}{{UAL}_{i}},$$

where Q_*i*_ stands for the sub-index parameter; n is the number of parameters taken for analysis; w_*i*_ depicts the weight of each parameter, evaluated as 1/*S*_***i***_; S_*i*_ symbolize the standard value of each parameter; Q_*i*_ represents the sub-index of the boundary. C_*ave*_ signifies the average concentration of each studied HMs; UAL_*i*_ stands for the upper allowable limit of the *i*th metal in the sample.

A three-class modified scale is frequently used to depict moderate levels of heavy metal pollution accurately. This scale classifies heavy metal pollution as very low (HPI < 25), low (26 ≤ HPI ≤ 50), medium (51 ≤ HPI ≤ 75), High (76 ≤ HPI ≤ 100), unsuitable (HPI > 100)^41^.

Metal index (MI) has six classes: very clean (MI < 0.3); clean (0.3 < MI < 1); partly affected (1 < MI < 2); moderately affected (2 < MI < 4); heavily affected (4 < MI < 6); and severely affected (MI > 6)^42^.

### Human health risk

The health risk assessment in this study adheres to the model recommended by the United States Environmental Protection Agency. This analysis explores the impact of environmental pollutants on health, categorizing risks into two main types: Carcinogenic (CR index) and non-carcinogenic (HQ and HI indices)^43^. Carcinogenic risks focus on the probability of developing cancer due to prolonged exposure to pollutants or a combination thereof. On the other hand, non-carcinogenic risks encompass various exposure-related effects, including genetic and teratogenic outcomes. Heavy metals (HMs) in drinking water enter the body primarily through ingestion and skin contact^44^. Consequently, this study's health risk assessment considers risks associated with direct drinking and skin contact, which are quantified through Eqs. (7) and (8).

8 $${\text{CDI}}_{\text{oral}}=\frac{{\text{C}}_{\text{w}}\times \text{IR}\times \text{EF}}{\text{BW}\times \text{AT}} \times \text{ED },$$

9 $${\text{CDI}}_{\text{dermal}}=\frac{{\text{C}}_{\text{ave}}\times \text{ET}\times \text{EF}\times \text{Kp}\times \text{SA}\times \text{CF}}{\text{BW}\times \text{AT}} \times \text{ED },$$

In the given equations, CDI oral represents the average daily direct intake dose, while CDI Dermal corresponds to the average daily dose absorbed by the skin. Cw signifies the concentration of heavy metals (HMs) in the water sample (mg/L), IR denotes the daily ingestion rate (L/d), EF stands for the exposure frequency, ED represents the exposure duration, BW indicates the body weight, SA is the exposed skin area, Kp is the skin permeability coefficient, CF is the conversion factor, and ET signifies the exposure time. For detailed values of these exposure parameters, refer to Table 1s. The calculation Hazard quotient (HQ), reference dose (RFD), Health risk index (HI), and Carcinogenic risk (CR) are reported in Eqs. (9), (10), (11) and (12), while the used parameters for calculation of health risk indices based on USEPA were added Table 1s.

10 $${HQ}_{dermal/oral}=\frac{{\text{CDI}}_{\text{dermal}}{/\text{CDI}}_{\text{oral}}}{{\text{RfD}}_{\text{dermal}}{/\text{RfD}}_{\text{oral}}} ,$$

11 $${RfD}_{dermal}={RfD}_{oral}\times ABS ,$$

12 $$HI=\sum HQ$$

The reference dose is denoted as RfD, and ABS signifies the digestive coefficient of the gastrointestinal tracking (as outlined in Table 1). The carcinogenic risk (CR) resulting from both direct ingestion and skin contact can be formulated as follows:

**Table 1:** The heavy metal concentrations in mg/l in the GOS water samples.

Sample	Cd	Cr (VI)	Cu	Fe	Mn	Ni	Pb	Zn
1	0.002	0.001	0.012	0.010	0.002	0.001	0.006	0.143
2	0.003	0.002	0.241	0.020	0.002	0.001	0.999	0.013
3	0.001	0.003	0.034	0.021	0.021	0.021	0.006	0.070
4	0.006	0.001	0.006	0.022	0.002	0.001	0.484	0.009
5	0.004	0.005	0.006	0.021	0.005	0.001	0.005	0.018
6	0.003	0.010	0.073	0.028	0.002	0.012	0.408	0.089
7	0.008	0.001	0.108	0.010	0.005	0.033	0.004	0.037
8	0.009	0.009	0.118	0.020	0.013	0.011	0.003	0.068
9	0.004	0.010	0.009	0.011	0.024	0.002	0.009	0.008
10	0.007	0.139	0.006	0.013	0.002	0.001	0.144	0.019
11	0.011	0.180	0.006	0.513	0.034	0.032	0.019	0.039
12	0.008	0.095	0.007	0.042	0.012	0.012	0.008	0.019
13	0.033	0.095	0.006	0.014	0.022	0.002	0.018	0.011
14	0.003	0.072	0.006	0.013	0.032	0.005	0.002	0.012
15	0.023	0.119	0.006	0.015	0.002	0.002	0.017	0.015
16	0.029	0.169	0.006	0.012	0.021	0.003	0.009	0.012
17	0.013	0.048	0.007	0.012	0.003	0.003	0.008	0.009
18	0.016	0.079	0.115	0.010	0.003	0.003	0.017	0.017
Min	0.001	0.001	0.006	0.010	0.002	0.001	0.002	0.008
Max	0.033	0.180	0.241	0.513	0.034	0.033	0.999	0.143
Mean	0.011	0.061	0.051	0.066	0.012	0.009	0.158	0.038

13 $$\text{CR}=\text{CDI }\times \text{ CSF} .$$

CSF is a conversion slope factor of metals, as detailed in Table 1.

### Monte Carlo simulation

Monte Carlo simulation is a strategy utilized in risk assessment to minimize uncertainties linked to heavy metal (HM) concentrations and exposure parameters, allowing for predicting both carcinogenic and non-carcinogenic risks. This method enables researchers to generate estimates of health risk values^8,29^. Typically, Python software, version 3.9.7, executes Monte Carlo simulations. In this study, the Python code underwent 10,000 iterations, facilitating Monte Carlo simulations to determine the probability risks related to carcinogenic and non-carcinogenic effects of heavy metals for both adults and children.

### Statistical analysis and pollution source identification

#### Cluster analysis

Cluster analysis (CA) is a valuable technique employed for pattern recognition in datasets from diverse sources without supervision. It identifies distinctive features that differentiate groups within the dataset and clusters them accordingly. R mode and Q mode approaches are used to execute and construct CA, aiding in creating clusters of water samples based on their chemical properties. CA is particularly useful in surface water studies, helping group collected water samples into meaningful geological and hydrogeological categories. The clustering process is often represented using a cluster dendrogram, simplifying the complexity of the data and providing a clear depiction of groupings and their relationships^8,23,27,45^.

#### Principal component analysis

Principal component analysis (PCA) is a linear technique that handles complex multivariate datasets. Its objective is to analyze data without losing information, summarize the dataset, and estimate the number of variables required to explain the variance. By reducing data dimensionality, PCA reveals hidden patterns and relationships between variables that may not be immediately apparent. The Kaiser Criterion uses eigenvalues from the scree plot to assess surface water contamination to extract principal components. Tests like Kaiser Meyer Olkin (KMO) and Bartlett's tests are employed to evaluate the suitability of data for factor analysis, indicating whether variables are adequate or inadequate within the model. KMO values falling within specific ranges indicate the appropriateness of data, ensuring that researchers gain meaningful insights from complex datasets. This comprehensive approach enhances understanding while providing the data accurately representing underlying relationships^9,23–28^.

## Results and discussion

### Water quality associated with PTEs

The concentrations of potential toxic elements (heavy metals) in the water samples reported in Table 1 were, on average, as follows: Cd (0.011 mg/L), Cr (0.061 mg/L), Cu (1.051 mg/L), Fe (0.006 mg/L), Mn (0.012 mg/L), Ni (0.009 mg/L), Pb (0.158 mg/L), and Zn (0.038 mg/L). These levels are arranged in descending order: Pb > Fe > Cr (VI) > Cu > Zn > Mn > Cd > Ni. Notably, the average concentrations of Cd, Cr (VI), and Pb exceeded WHO's standard limits for drinking purposes^48^, while the concentrations of the other heavy metals remained within acceptable limits. This highlights a concerning contamination, especially for Cd, Cr, and Pb. Effective management strategies are crucial to address this issue, ensuring the quality of water resources and protecting public health. Increased monitoring and regulatory measures should be implemented, specifically focusing on the heavy metals that surpass recommended standards, to guarantee the safety and sustainability of water sources in the studied area.

### Environmental and ecological risk

#### Pollution risk of PAHs on aquatic life and biodiversity

The studied PAHs were 16 compounds (Table 2): naphthalene (Naph), acenaphthylene (Acthy), acenaphthalene (Ace), fluorene (Fl), phenanthrene (Phe), anthracene (Ant), fluoranthene (Flu), pyrene (Pyr), benzo(a)anthracene (BaA), chrysene (Chry), benzo(b)fluoranthene (BbF), benzo(k)fluoranthene (BkF), benzo(a)pyrene (BaP), dibenzo(a,h)anthracene (DBA), benzo(g,h, i)perylene (BghiP) and indo(1,2,3-cd)pyrene (InP). The distribution of the 16 PAHs in the water was inconsistent, with pyrene (Pyr) having the most significant concentration (443 µg/L) in site No.1 (Suez) and acenaphthylene (Ace) having the lowest concentration (0.120 µg/L) at site No.3 (Northern Zaafarana). Pyr may be created when low aromatic PAH rings condense at high temperatures^17^.

**Table 2:** The PAHS concentrations in µg/L in the GOS water samples including the names of the main locations (Suez, Ain Sukhna, Zaafarana N, Zaafarana S, Ras Gharib, Gabal El Zeit, and Qusier).

Concentration (µg/L water)	No.1 (Suez)	No.2 (Ain Sukhna)	No.3 (Zaafarana N.)	No.4 (Zaafarana S.)	No.5 (Ras Gharib)	No.6 (Ras Shokier)	No.7 (Gabal El Zeit)	No.8 (Qusier)	Total
Naph	ND	0.084	1.77	0.855	0.041	0.102	0.088	1.50	4.44
Acthy	ND	ND	0.120	ND	ND	ND	ND	ND	0.120
Ace	1.22	ND	0.045	ND	ND	ND	ND	ND	1.26
Fl	ND	0.303	0.463	0.265	0.337	0.388	0.291	0.373	2.42
Phe	5.18	1.24	1.349	1.17	1.00	1.06	1.40	1.39	13.80
Ant	ND	0.229	ND	ND	ND	ND	ND	0.505	0.734
Flu	ND	0.519	0.217	0.803	0.412	0.205	0.297	0.190	2.64
Pyr	443	0.87	1.16	1.55	0.675	0.599	0.739	1.42	449.65
BaA	ND	ND	0.54	ND	ND	1.84	ND	0.173	2.55
Chry	ND	0.22	ND	0.532	ND	ND	0.150	0.346	1.25
BbF	2.12	1.79	2.56	1.41	0.396	2.36	0.179	1.13	11.96
BkF	8.04	1.72	12.37	0.635	1.09	4.85	4.28	3.86	36.86
BaP	19.46	3.79	1.86	3.06	0.294	3.76	2.65	5.14	40.01
DBA	ND	1.28	1.23	0.684	1.01	0.943	0.592	2.77	8.51
BghiP	ND	4.28	0.277	0.626	2.39	1.11	1.12	0.272	10.08
InP	ND	3.34	2.76	3.49	3.48	3.23	2.98	3.46	22.75
Total PAHs	479	19.68	26.73	15.08	11.14	20.45	14.78	22.54	609.07
Total CAR	29.62	16.44	21.61	10.44	8.67	18.09	11.96	17.15	133.98
LPAHs	6.41	1.85	3.75	2.29	1.38	1.55	1.78	3.77	22.79
HPAHs	472.25	17.83	22.98	12.79	9.76	18.90	13.00	18.76	586.28

The Suez area has the greatest concentration of PAHs in the Suez Gulf (479 µg/L), primarily due to the refineries and oil production areas that are close. Following, the concentrations in the northern part of Zaafarana (about 26.73 µg/L), Qusier area in the South (22.537 µg/L), Ras Shukeir (20.45 µg/L) and near Ain Sukhna Resort (19.685 µg/L) are approximately the same. The low concentrations at some southern locations might be attributed to the extended distance from the point source^15^. Generally, the total PAH concentrations at the eight locations are far greater than the maximum permissible concentrations of the European Union, which is 0.20 µg/L, and the Environmental Quality Criteria of the United States, which is 0.030 µg/L for the protection of human consumers of aquatic life^49^. The United States Environmental Protection Agency (USEPA) and the World Health Organization (WHO) classified the PAHs Naph, Acthy, Fl, Phe, Ant, Pyr, Flu, and BaP as dangerous pollutants (CAR) among the other PAHs^15,49,50^. CAR ranged from 8.67 µg/L at Ras Gharib to 29.62 µg/L at Suez.

#### Source identification of PAHs in GOS

The marine environment primarily encounters two significant sources of polycyclic aromatic hydrocarbons (PAHs): petrogenic and pyrogenic sources. Pyrogenic sources stem from the incomplete combustion of fossil fuels, while petrogenic sources originate from the natural release of petroleum or its derivatives into the environment. While the high molecular weight (HPAHs) PAHs have four to six rings and are highly carcinogenic, the low molecular weight (LPAHs) only have two to three rings. With LPAHs/HPAHs > 1 indicates petrogenic, while less than one indicates pyrogenic^15,17^. The ratios in the current seawater data for all 8 locations were less than 1, which suggests a pyrogenic origin for the majority of the PAHs.

In pyrogenic assemblages, the Flu/Pyr ratio often reaches or rises above a value of one, whereas in petrogenic PAH assemblages, it usually drops below one. APhen/Ant varied from 5.406 in site No. 2 (Ain Sukhna) to 2.76 in site No.8 (Qusier). This refers to a pyrogenic nature for both (< 10), which agrees with the conclusion taken from the above LPAHs/HPAHs ratios. Meanwhile, site No.8 (Qusier) was supposed to come from a pyrogenic source (< 5), and site No. 2 (Ain Sukhna) was petrogenic in origin (> 5). Site No. 8 (Qusier) showed a BaA/(BaA + Chr) ratio that was slightly less than 0.35 and more than 0.2 (0.333), suggesting different sources because of industrial and shipping activity. Conversely, all locations displayed Flu/Pyr ratios of less than one and Flu/(Flu + Pyr) ratios of less than 0.4, indicating the presence of petroleum sources there^15,17^.

The comparison of PAH levels along the GOS (Red Sea) with those reported worldwide (Table 4) provides valuable insights into the current pollution status. Efforts were made to select references similar to our study for a meaningful comparison. Total PAH levels (μg/L) in various locations, including the Mississippi River, Susquehanna River in the USA, Seine River in France, Surface waters, Hangzhou, Rivers in Tianjin, The Yellow River in China Giresun Coast, Samsun Coast in Turkey were observed to be lower than those recorded in the present study. This indicates relatively lower PAH pollution levels in these areas compared to the Red Sea in Egypt (GOS) (Table 3). The comparison of PAH levels along the GOS (Red Sea) with those reported worldwide (Table 4) offers critical insights into the current pollution status. It underscores the need for comprehensive risk assessment and mitigation strategies. A meaningful comparison was made possible by carefully selecting references akin to our study. It is noteworthy that total PAH levels (μg/L) in various locations, including the Mississippi River, Susquehanna River in the USA, Seine River in France, Surface waters in Hangzhou, Rivers in Tianjin, The Yellow River in China, as well as the Giresun and Samsun Coasts in Turkey, were observed to be lower than those recorded in the present study along the GOS. This disparity suggests a heightened level of PAH pollution in the GOS region, warranting urgent attention and proactive measures to mitigate environmental and public health risks. In interpreting these findings, it becomes evident that the elevated PAH levels in the GOS could have significant ecological ramifications, including adverse effects on marine biodiversity and ecosystem functioning. Moreover, considering the potential bioaccumulation of PAHs in aquatic organisms and their subsequent transfer along the food chain, there may be implications for human health, particularly for communities reliant on marine resources for sustenance. To address these concerns, it is imperative to conduct robust risk assessments that encompass both ecological and human health dimensions. Such assessments should evaluate the sources, pathways, and fate of PAHs in the marine environment and their potential impacts on vulnerable species and human populations. Additionally, targeted monitoring programs should be established to track PAH levels over time and assess the effectiveness of pollution control measures. Based on the outcomes of these risk assessments, tailored mitigation strategies should be developed and implemented, focusing on reducing PAH emissions from industrial and anthropogenic sources, enhancing wastewater treatment infrastructure, and promoting sustainable land use practices. Public awareness campaigns and stakeholder engagement initiatives can also be crucial in fostering a culture of environmental stewardship and community resilience to PAH pollution.

**Table 3:** The TOC concentrations in µg/L in the GOS water samples including the names of the main locations (Suez, Ain Sukhna, Zaafarana N, Zaafarana S, Ras Gharib, Gabal El Zeit, and Qusier).

Location	TOC (mg/L)
No.1 (Suez)	2.31
No.2 (Ain Sukhna)	1.64
No.3 (Zaafarana N.)	1.52
No.4 (Zaafarana S.)	1.52
No.5 (Ras Gharib)	1.57
No.6 (Ras Shokier)	1.54
No.7 (Gabal El Zeit)	1.63
No.8 (Qusier)	1.63

**Table 4:** The comparison of PAH levels along the GOS (Red Sea) with those reported worldwide.

Country	Water type	Number of samples	∑PAHs range value (μg/L)	Reference
USA	Mississippi River	4	0.006–0.069	^51^
USA	Susquehanna River	36	0.017–0.15	^52^
France	Seine River	6	0.004–0.036	^53^
China	Surface waters, Hangzhou	17	0.989–9.663	^54^
China	Rivers in Tianjin	10	0.058–1.060	^55^
China	The Yellow River	26	0.144–2.361	^56^
Turkey	Giresun Coast	16	0.04742–0.57669	^57^
Turkey	Samsun Coast	16	0.072–1.186	^58^
Turkey	Giresun Coast	16	0.049–0.444	^59^
Egypt	Red sea	8	11.14—479	

#### TOC (total organic carbon)

The mean TOC concentration was 1.671 mg/L, ranging from 1.52 to 2.31 mg/L. Because of the decreased frequency of tidal floods in this semi-enclosed basin and the higher deposition of debris on the surface of the sediments, the Suez Bay region had the highest value for TOC. Additionally, oil rigs and refineries significantly impact this area. They lead to oil spills in a constrained environment and deteriorate water quality by a high percentage of organics.

#### Potential ecological risks of PAHs

The value of RQ was used to classify the risk grade (Table 2s) of individual and total PAHs. For total PAHs, the ecological risk of surface seawater (Table 5) was at a high-risk level in sites NO.1, 3, and 8 with RQ_NCs_ ≥ 800 and RQ_MPCs_ ≥ 1.0 and medium risk 2 with RQ_NCs_ < 800 and RQ_MPCs_ ≥ 1.0 in site No.2, 4, 6 and 7. Furthermore, total PAHs were more predictable to pose a concern to the environment in Suez than in the other sites. Except for one or two sites, the RQ_NCs_ of Acthy and Ace were < 1.0, indicating that the ecological risk of Acthy and Ace was insignificant in the surface saltwater of the GOS coast. Apart from Acthy and Ace, all other PAH compounds had RQMPCs greater than 1.0, indicating that 11 of the 16 PAHs posed a significant danger (Table 3). Furthermore, all 16 PAHs were most likely to cause harmful ecological effects with extremely high RQ_NCs_. In summary, PAHs in surface seawater from the Red Sea's uppermost portion and the coastal shores of the GOS pose significant ecological harm to aquatic life^49^.

**Table 5:** Potential ecological risks of PAHs represented by RQ values.

PAHs	Water (µg/L)		No.1 (Suez)		No.2 (Ain Sukhna)		No.3 (Zaafarana N.)		No.4 (Zaafarana S.)		No.5 (Ras Gharib)		No.6 (Ras Shokier)		No.7 (Gabal El Zeit)		No.8 (Qusier)	
	NCs	MPCs	RQ_NCs_	RQ_MPCs_	RQ_NCs_	RQ_MPCs_	RQ_NCs_	RQ_MPCs_	RQ_NCs_	RQ_MPCs_	RQ_NCs_	RQ_MPCs_	RQ_NCs_	RQ_MPCs_	RQ_NCs_	RQ_MPCs_	RQ_NCs_	RQ_MPCs_
Naph	12	1200	0	0	7	0.07	147.5	1.5	71.3	0.7	3.4	0.03	8.5	0.08	7.3	0.07	125.16	1.2
Acthy	0.7	70	0	0	0	0	17.1	0.2	0	0	0	0	0	0	0	0	0	0
Ace	0.7	70	1744.2	17.4	0	0	64.3	0.6	0	0	0	0	0	0	0	0	0	0
Fl	0.7	70	0	0	432.8	4.3	661.4	6.6	378.5	3.8	481.4	4.8	554.3	5.5	415.7	4.2	532.8	5.3
Phe	3	300	1729	17.2	412.6	4.1	449.6	4.4	390	3.9	334.6	3.3	353	3.5	467.3	4.7	465.3	4.6
Ant	0.7	70	0	0	327.1	3.2	0	0	0	0	0	0	0	0	0	0	721.4	7.2
Flu	3	300	0	0	173	1.7	72.3	0.7	367.6	3.7	137.3	1.4	68.3	0.7	99	0.9	63.3	0.6
Pyr	0.7	70	632,344.2	6323.4	1245.7	12.4	1654.2	16.5	2220	22.2	964.3	9.6	855.7	8.6	1055.7	10.5	2027.1	20.3
BaA	0.1	10	0	0	0	0	5440	54.4	0	0	0	0	18,380	183.8	0	0	1730	17.3
Chry	3.4	340	0	0	65.5	0.6	0	0	156.5	1.6	0	0	0	0	44.1	0.4	101.7	1.01
BbF	0.1	10	21,170	211.7	17,990	179.9	25,620	256.2	14,090	140.9	3960	39.6	23,620	236.2	1790	17.9	11,350	113.5
BkF	0.4	40	20,092.5	200.9	4310	43.1	30,937.5	309.4	1587.5	15.8	2727.5	27.3	12,127.5	121.3	10,717.5	107.2	9657.5	96.5
BaP	0.5	50	38,922	389.2	7580	75.8	3722	37.2	6116	61.2	588	5.8	7520	75.2	5308	53.08	10,274	102.7
DBA	0.5	50	0	0	2572	25.7	2452	24.5	1368	13.7	2024	20.2	1886	18.8	1184	11.8	5536	55.3
BghiP	0.3	30	0	0	14,263.3	142.6	923.3	9.2	2086.6	20.8	7983.3	79.8	3710	37.1	3740	37.4	906.6	9.1
InP	0.4	40	0	0	8347.5	83.4	6905	69.05	8730	87.3	8717.5	87.2	8075	80.7	7452.5	74.5	8645	86.4
Total PAHs	27.2	2720	17,597.9	175.9	723.7	7.2	982.7	9.8	554.5	5.5	409.6	4.09	751.8	7.5	543.4	5.4	828.5	8.3

#### Possible ecological risks of PAHs on biodiversity

The detrimental effects of PAHs on aquatic creatures in natural habitats have been documented in earlier studies. The biomagnification of PAHs may adversely impact the productivity and health of the marine environment through the food chain (PAH troph), which may also harm the health of people who eat these aquatic species. PAH consumption is linked to an increased risk of cancer, birth abnormalities, and mutations. Even though HMW-PAHs have more significant carcinogenic risks than PAHs with smaller ring systems, fluoranthene is light-induced toxicity to coral. In fact, BaP toxicity is used in human health risk assessment to show the carcinogenic potential risk of PAH contamination with the intake of coral reef fish. Since BaP is the only PAH for which toxicological data network (TOXNET) are sufficient for the generation of a cancer potency factor (CPF) among all known other PAHs that may cause cancer, certain regulatory authorities have established a standard for quantitative risk assessment of PAHs (particularly for BaP) in food^60,61^. High levels of PAHs in fish livers posed a concern to the health of coral fish; they also disrupted the energy metabolism of Mytilus galloprovincialis^60^. Additionally, it has been suggested that PAH may interact with DNA through the BaPDE's binding to the nucleophilic centers of exocyclic amino groups of purines to form DNA adducts or other genotoxic effects when it is converted to the diol epoxides of BaPDE (benzo[a] pyrene-7,8-diol-9,10-epoxide)^61^. Coral reefs, in particular, are sensitive to oil spills because of the rugosity and porosity of their structures, which allow oil to be stored for extended periods. As a result, coral populations suffer from acute to chronic impacts, affecting reproduction and maintenance throughout time^62^. Significant amounts of organic substances or mixtures (for example, crude oil) can poison corals by altering their development, recruitment, and reproduction, resulting in substantial mortality rates for colonies. Regrettably, even modest quantities of PAHs in the environment can be rapidly absorbed and stored in corals^63^. Furthermore, the negative impacts of these PAHs on corals have been demonstrated. For example, benzo[a]pyrene (BaP) was found to decrease antioxidant enzyme activity in coral embryos, generating significant alterations in gene-expression pathways. It was shown that the PAH anthracene (Ant), which accumulates readily in cell membranes and alters cellular metabolism, may impede coral larval settling and survival. The combined interaction of UV light and fluoranthene (Flu) in coral reefs may result in 78% mortality and bleaching^64^.

Oil pollution also has an impact on mangrove trees, which continue to play an important role in maintaining ecological balance by giving nutrients to the maritime environment and forming forests of salt-tolerant plant species that contain a large number of marine microorganisms. Organic pollutants in mangroves, like as polycyclic aromatic hydrocarbons, have been a major source of worry due to their persistence, bioaccumulation, toxicity, and long-range transmission. This kind of pollution prevents growth, lessens photosynthesis, causes leaf necrosis, and eventually causes death. Mangrove roots have a larger abundance of HMW PAHs than LMW PAHs. This is because LMW PAHs can move from roots to shoots, but HMW PAHs are only weakly translocatable and firmly adsorbed in the roots. Through slow diffusion from the root surface via plasmodesmata, PAHs enter the tissues. Being hydrophobic, PAHs are bound to the lipid portions of the organelles and cell membranes after they have entered the tissues. By increasing the permeability of lipidic molecules, PAHs harm cell membranes. Additionally, the chloroplasts, mitochondria, and nuclei of cells are disrupted and fragmented by these organic contaminants, which can result in cell death. PAHs disrupt the regulation of the trans membrane electrical potential, which governs metal absorption and bioavailability, by harming the cell membrane. PAHs appear to impede metallothioneins, which play an important role in metal detoxification. Contamination of PAHs in mangrove sediments may potentially encourage unfavorable genetic changes in mangrove plants^6–65^.

#### Ecological risk of PTEs in coastal water

**Fig. 2:**
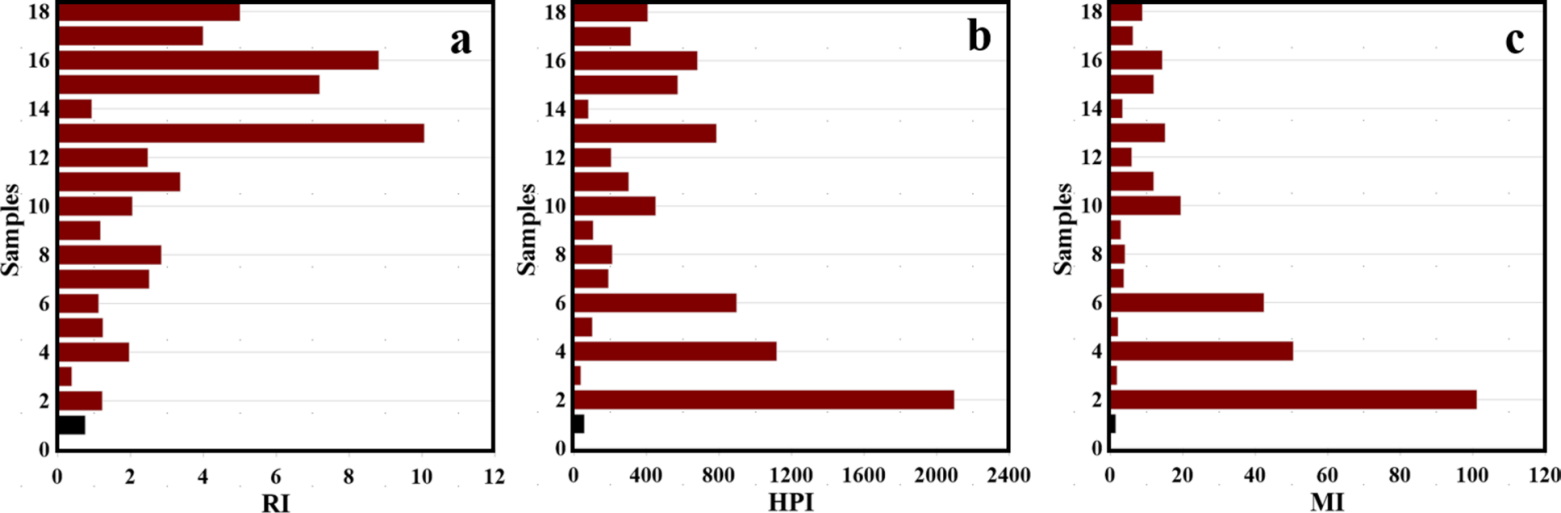
The ecological risk index (**a**), heavy metals pollution index (**b**), and metal index (**c**) calculated from 18 locations of the GOS.

The ecological risk index (RI) is a crucial tool incorporating diverse factors like heavy metal concentration, ecological impact, toxicology, and environmental consequences. This enables the assessment of ecological risks linked to heavy metal pollution in both sediment and water. In this study, the investigation into the potential environmental risk index of PTEs in the Red Sea, as depicted in Fig. 2a, revealed an average RI of 3.38 and a maximum value of 10.06 for the collected samples. This suggests a relatively low ecological risk (RI < 30). The visualization in Fig. 2a establishes a connection between pollution levels and ecological risk across different locations in the study area. Notably situated near residential areas and petroleum extraction sites, the study area may contribute to heightened concentrations of PTEs in the Red Sea. Thus, a comprehensive assessment of the ecological risk associated with heavy metal contamination in aquatic ecosystems becomes imperative. This research plays a vital role in guiding effective management strategies and mitigating potential risks these trace pollutants pose.

#### Heavy metals pollution index and metal index

The HPI for studied heavy metal elements in the Red Sea by 2023 has been recorded, with a mean HPI value of 537.8 and a range of 38.02 to 2097.8 (Fig. 2b). According to Ref.^41^, almost 5.55%, 5.55%, 5.55%, and 83.33% of samples fall within the Good, poor, very poor, and unsuitable class, respectively, based on the HPI rating, indicating minimal contamination in surface water only in sample 1 while the other location is highly contaminated. To understand the impact of heavy metals on water quality, the MI method, alongside the HPI index, assessed the level of contamination based on the upper allowable limit values of the WHO guideline^48^. The average MI values were 20.5 and ranged from 1.5 to 101.2, indicating variation in the contamination degree with different locations (Fig. 2c). It was found that 11.11%, 22.22%, 11.11%, and 55.55% of samples fall within the partly affected, moderately affected, heavily affected, and severely affected class based on the MI rating, indicating minimal contamination in surface water only in sample 1 and sample 3 while the other location is highly contaminated.

### Human health risk

This section focuses on evaluating health risks associated with exposure to various heavy metals through both ingestion and dermal absorption pathways. The assessment involves calculating hazard quotients (HQ) for non-carcinogenic and carcinogenic risks using hazard indices (HI). The results provide insights into the overall potential health risks for individuals, considering both children and adults, who may come into contact with these heavy metals. The emphasis is on understanding the combined impact of different exposure routes on human health.

#### Non-carcinogenic risk

**Fig. 3:**
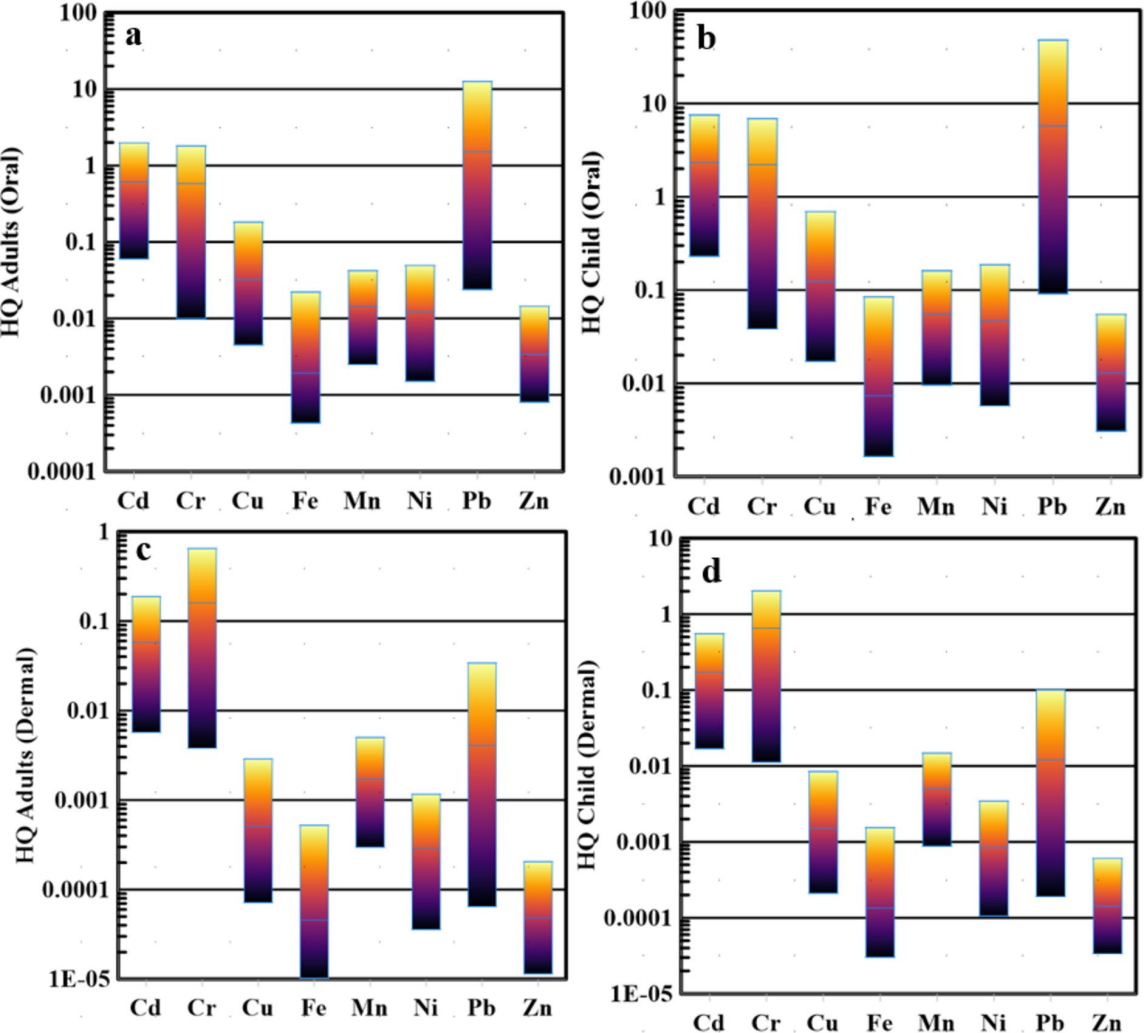
Box plot of HQ oral in adult (**a**), child (**b**), HQ dermal in adult (**c**), and child (**d**).

The toxic elements cadmium, chromium, copper, iron, manganese, nickel, lead, and zinc were evaluated to determine the non-carcinogenic risk in children and adults (Fig. 3a–d). It's crucial to emphasize that the HQ oral values for both children and adults fall within the acceptable limit (below 1) for copper (Cu), iron (Fe), manganese (Mn), nickel (Ni), and zinc (Zn). However, in specific instances, the HQ oral values for cadmium (Cd), chromium (Cr), and lead (Pb) surpass 1, indicating an elevated risk. Specifically, the HQ oral value for adults exceeded 1 in 16.7%, 22.22%, and 22.22% of the water samples for Cd, Cr, and Pb, respectively. Similarly, the HQ oral value for children exceeded 1 in 61.11%, 50%, and 22.22% of the water samples for Cd, Cr, and Pb, respectively. These percentages are tied to the location and timeframe of the study, underscoring the necessity of considering diverse factors like exposure duration and frequency, individual susceptibility, and environmental conditions in evaluating the actual human health risks. The hazard quotient (HQ) dermal values indicate that the potential health risks associated with dermal exposure to heavy metals are generally within acceptable limits for adults, staying below the threshold of 1 for all parameters. Conversely, the HQ dermal value for children exceeded 1 in 33.33% of the water samples for chromium (Cr) while remaining within acceptable limits for the other heavy metals. This highlights a concern for children exposed to Cr through dermal contact in a third of the studied water samples. As with oral exposure, these findings emphasize the importance of location-specific considerations and the need to account for various factors such as individual susceptibility, environmental conditions, and the duration and frequency of exposure when assessing actual human health risks. The results suggest that Cd, Cr, and Pb are the primary contributors to human health risks for both adults and children, whether through oral or dermal contact. Notably, in the case of dermal exposure, children exhibit increased vulnerability to chromium (Cr) from the coastal waters of the Red Sea. At the same time, adults face no significant risk through this route.

**Fig. 4:**
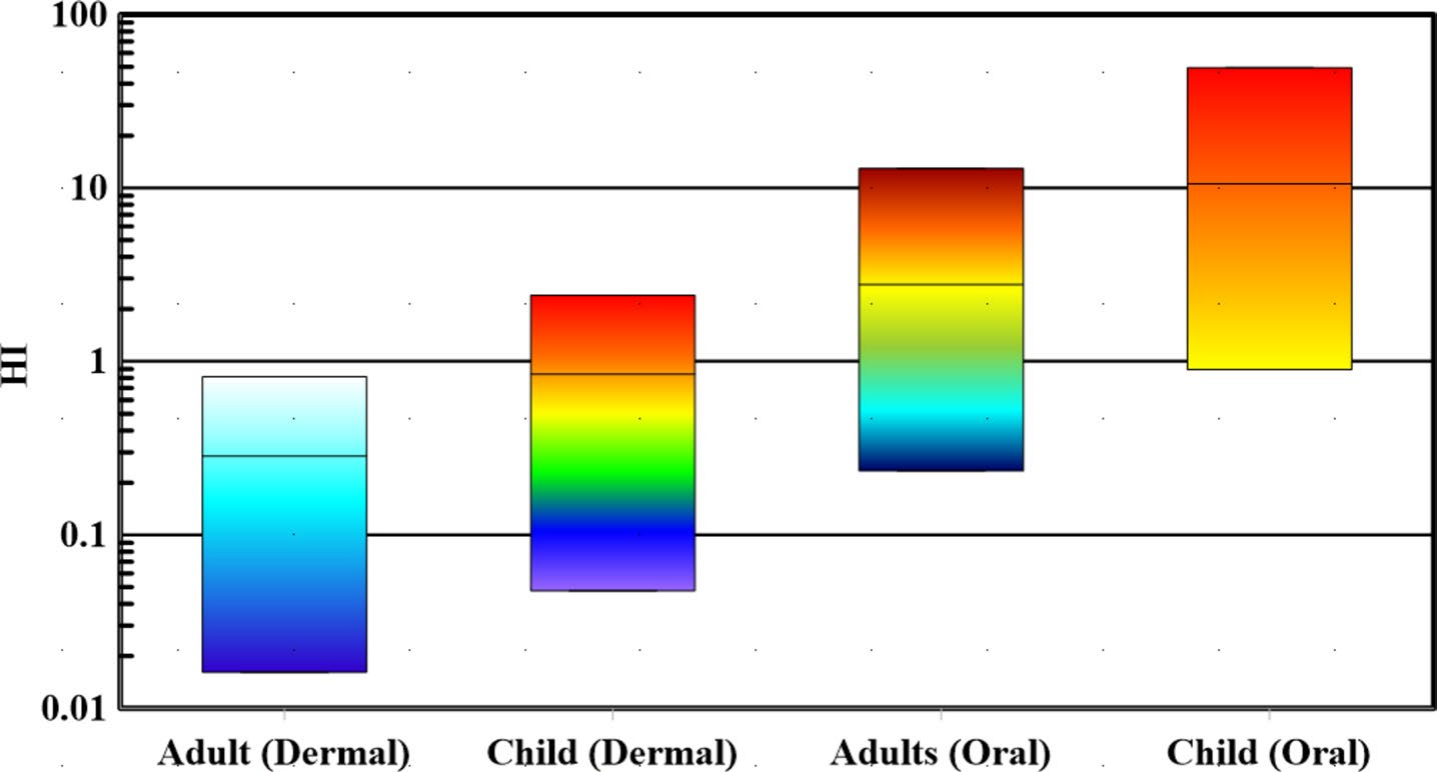
Box plot of HI (oral & dermal) index in children and adults.

The hazard index (HI) is a vital tool for gauging the overall health risks linked to heavy metals in Siwa Oasis's surface water. It considers various exposure routes, including ingestion and dermal pathways, offering a comprehensive view of combined health hazards from heavy metal contamination. Calculated by summing Hazard Quotients (HQs) for each heavy metal and exposure route, HI values (Fig. 4) ranged from 0.23 to 12.94 for adults and 0.89 to 49.39 for children through oral exposure. HI values ranged from 0.02 to 0.81 for adults and 0.05 to 2.4 for children for dermal exposure. Analysis revealed that HI oral values for adults and children exceeded safe levels (HI > 1) in 61.11% and 88.88% of water samples, respectively, indicating a high-risk category for non-carcinogenic impact. Conversely, HI values for adults indicated that 100% of water samples fell within the low-risk category for dermal contact. However, HI values for children showed 61.11% in the low-risk class and 38.88% in the high-risk category for dermal contact. This study underscores children's vulnerability to oral and dermal exposure to heavy metals, stressing the need to monitor these metals in the untreated water of the Red Sea. With the high cost of treating and desalinating saltwater, a primary water resource due to the study area's distance from primary freshwater sources like the Nile River, understanding and mitigating potential health risks is crucial. Specifically, samples 10, 11, 12, 13, 15, 16, and 18 were identified as the most contaminated, posing notable non-carcinogenic risks based on the HI (dermal and oral).

#### Carcinogenic risk

**Fig. 5:**
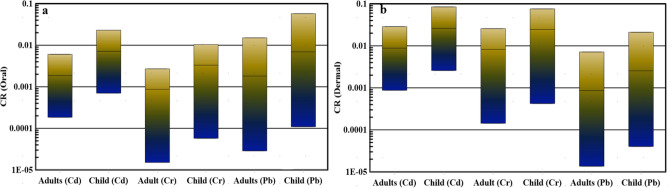
Box plot of CR (children and adults) index through oral contact (**a**) and dermal contact (**b**).

Carcinogenic risks involve assessing the likelihood of developing cancer due to prolonged exposure to pollutants. Our study used the traditional Carcinogenic Risk (CR) calculation as a baseline and compared it with CR values obtained through Monte Carlo simulation. For adults, the CR oral values for cadmium (Cd), chromium (Cr), and lead (Pb) ranged between 0.0002 to 0.006, 0.00002 to 0.003, and 0.00003 to 0.02, respectively (Fig. 5a). For children, these values ranged from 0.0007 to 0.02, 0.00006 to 0.01, and 0.0001 to 0.06 for Cd, Cr, and Pb, respectively (Fig. 5a). Analyzing the oral contact with heavy metals from the Red Sea water, we found a high carcinogenic risk (CR > 1 × 10–4) in 100%, 66.66%, and 66.66% of water samples for Cd, Cr, and Pb, respectively, in adults. For children, this risk was identified in 100%, 83.33%, and 100% of water samples for Cd, Cr, and Pb, respectively. Moving on to dermal exposure, the CR dermal values for adults were between 0.0009 to 0.03, 0.0001 to 0.03, and 0.00001 to 0.007 for Cd, Cr, and Pb, respectively (Fig. 5b). For children, these values ranged from 0.003 to 0.08, 0.0004 to 0.08, and 0.00004 to 0.02 for Cd, Cr, and Pb, respectively (Fig. 5b).

Examining dermal contact with heavy metals from the water samples revealed a high carcinogenic risk (CR > 1 × 10–4) in 100%, 100%, and 44.44% of water samples for Cd, Cr, and Pb, respectively, in adults. For children, this risk was found in 100%, 100%, and 83.33% of water samples for Cd, Cr, and Pb, respectively. These results underscore the urgent need for an effective treatment technique for the coastal water of the Red Sea before using it for any purpose. Regular monitoring of heavy metal concentrations after desalination is crucial, given the substantial carcinogenic risk to the health of both children and adults.

### Monte Carlo simulation

**Fig. 6:**
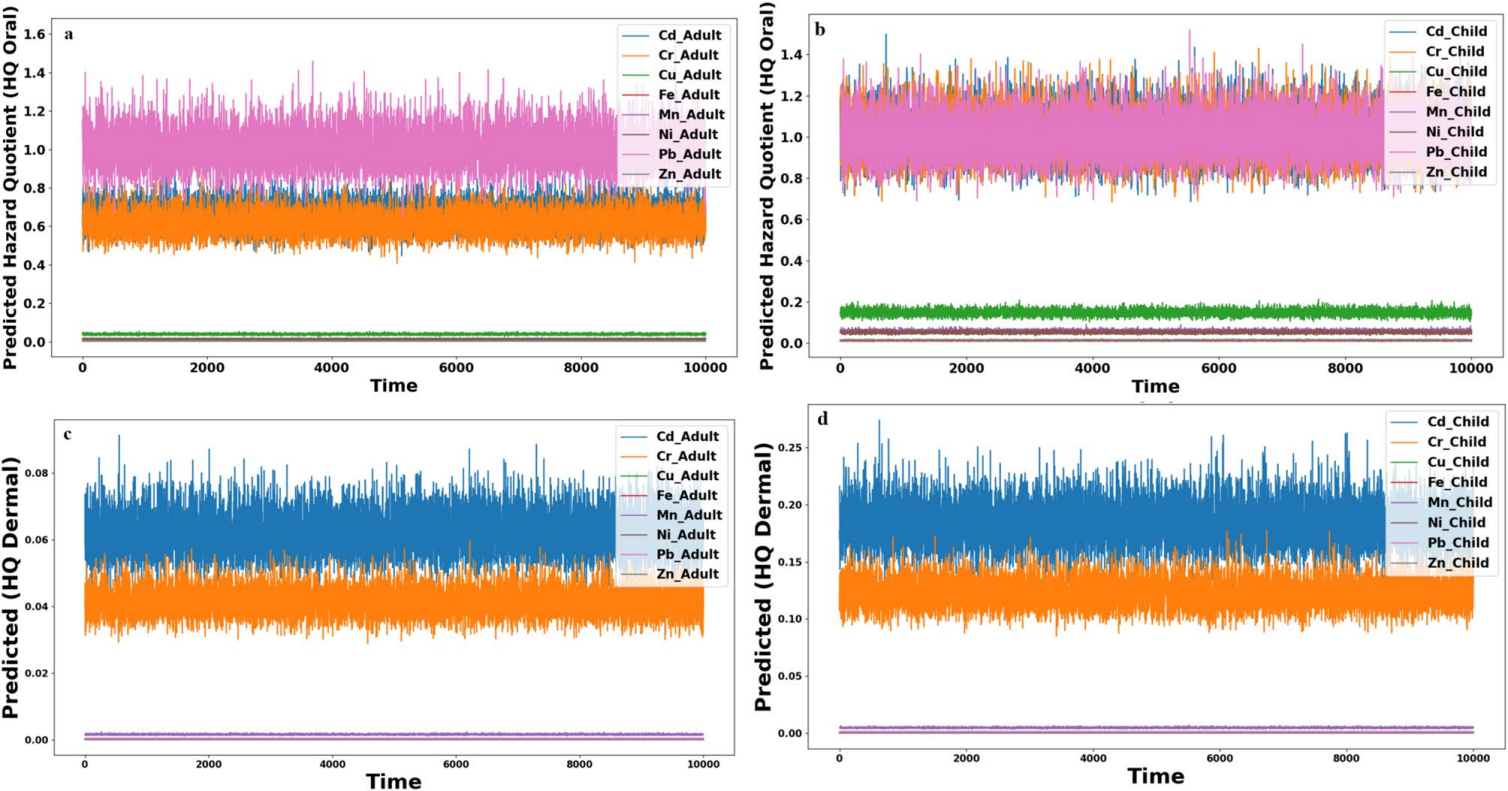
The predicted HQ oral in adult (**a**), child (**b**), HQ dermal in adult (**c**), and child (**d**).

**Fig. 7:**
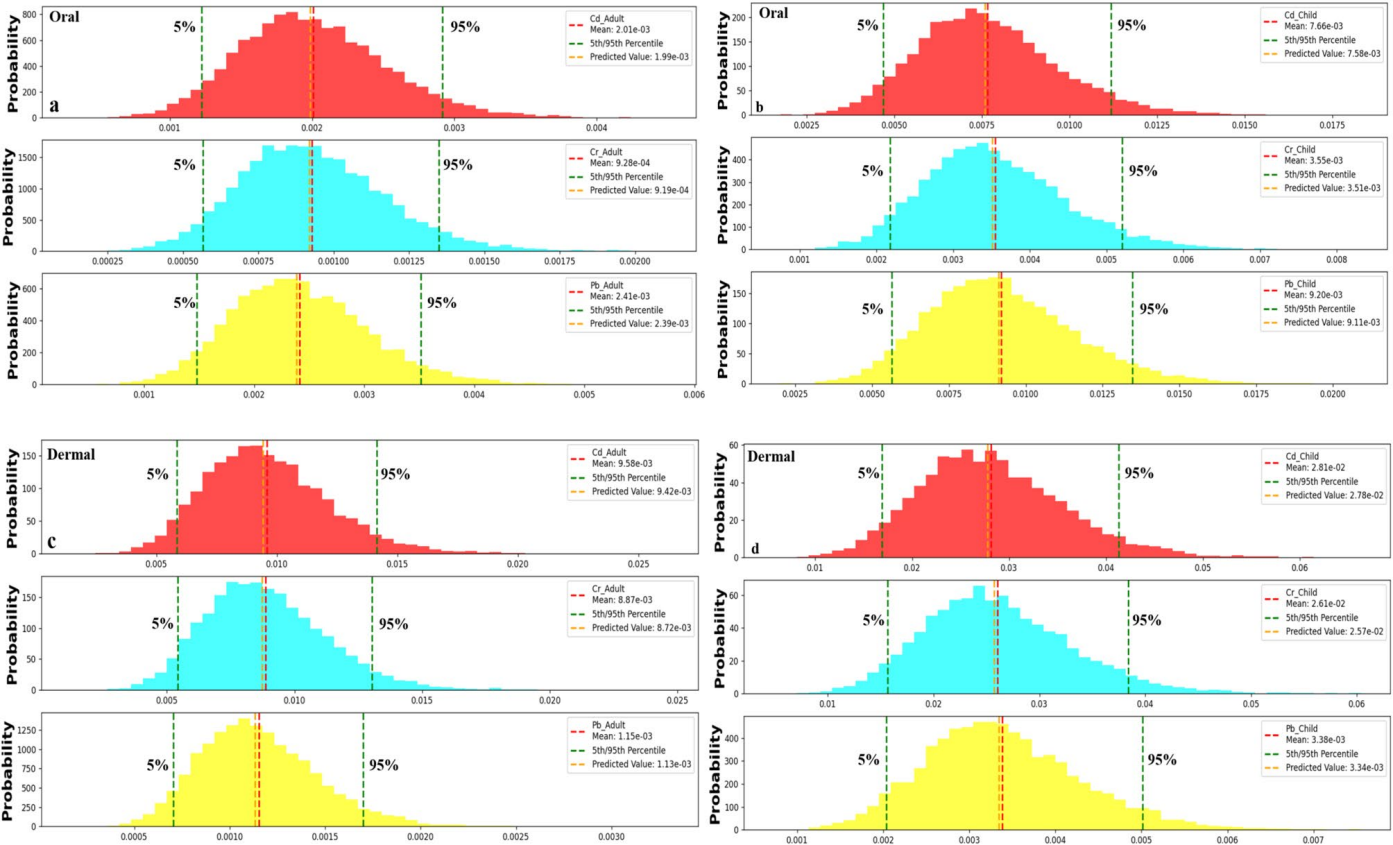
The predicted CR oral value with 5% and 95% of the datasets in adult (**a**), child (**b**), HQ oral in adult (**c**), and child (**d**).

The Monte Carlo simulation, a statistical tool, anticipates health risks from eight heavy metals (cadmium, chromium, copper, iron, manganese, nickel, lead, and zinc). It estimates HQ (Fig. 6) for non-carcinogenic risks through oral and dermal exposure and CR for cancer-related concerns in cadmium, chromium, and lead (Fig. 7). HQ reveals potential health impacts, while CR assesses cancer risks. Tailored for adults and children, considering age-specific susceptibilities, this simulation enhances our understanding of heavy metal health risks. The predictions contribute vital insights for practical risk assessment and management strategies, which are crucial in safeguarding human health from environmental threats.

#### Non-carcinogenic risk

The Monte Carlo simulation results provide valuable insights into the non-carcinogenic health risks associated with heavy metal exposure through oral and dermal pathways. For adults (Fig. 6a), the HQ oral values were generally below 1, indicating low non-carcinogenic risk for most metals except for Pb, which showed a higher risk. In contrast, children exhibited higher non-carcinogenic risks (HQ oral > 1) for Cd, Cr, and Pb (Fig. 6b), highlighting potential health concerns in this age group. Interestingly, the predicted HQ dermal values for adults and children were consistently below 1 for all eight metals, suggesting dermal exposure poses a lower non-carcinogenic risk. This finding is reassuring, indicating that when in contact with the skin, the water samples do not pose a significant threat regarding non-carcinogenic health risks for adults and children (Fig. 6c,d),.

Comparing the Monte Carlo results with traditional calculations, the simulation method proved effective in reducing uncertainty and providing realistic estimates. The confirmation of previous calculations adds robustness to the study's findings, emphasizing the reliability of the Monte Carlo simulation in assessing non-carcinogenic health risks associated with heavy metal contamination in water samples. This contributes to understanding potential health risks and supports informed decision-making for water quality management.

#### Carcinogenic risk

##### Oral exposure routes

The assessment of carcinogenic risk (CR) from oral exposure to heavy metals in Red Sea water samples reveals distinct patterns between children and adults. Across all parameters, CR values consistently indicate a higher likelihood of cancer risk in children compared to adults. Adults demonstrated relatively lower estimated cancer risks. The 5th percentile CR oral values for adults were 0.0012, 0.00056, and 0.0014 for Cd, Cr, and Pb, respectively, with 95th percentile values of 0.0029, 0.0013, and 0.0035, indicating reduced potential risks compared to children (Fig. 7a). In contrast, children exhibited 5th percentile CR oral values of 0.005, 0.0022, and 0.0056 for Cd, Cr, and Pb, respectively, with elevated risks at the 95th percentile (0.011, 0.0052, and 0.0134) (Fig. 7b). Nevertheless, predicted CR through oral exposure suggests a high cancer risk in many Red Sea water samples for both children and adults (CR > 1 × 10^–4^). The comparison of calculated and predicted CR underscores the effectiveness of Monte Carlo simulation in providing accurate estimations of cancer risk, emphasizing the substantial carcinogenic impact of specific metals on both age groups. This highlights the urgency of implementing measures to mitigate heavy metal exposure in the coastal waters of the Red Sea, particularly in regions where risks are most pronounced.

##### Dermal exposure routes

Evaluating carcinogenic health risks through skin contact in children and adults uncovers notable trends. Children consistently show higher probabilities of cancer risk than adults across various parameters. For adults demonstrate lower estimated CR levels for cancer risk through dermal exposure, with 5th percentile values of 0.0058, 0.0054, and 0.0007 for Cd, Cr, and Pb, and 95th percentile CR dermal values at 0.014, 0.013, and 0.0017, respectively (Fig. 7c).

In contrast, children, the estimated 5th percentile CR levels for cancer risk through skin contact were 0.016, 0.015, and 0.002 for Cd, Cr, and Pb, respectively, with 95th percentile CR levels at 0.041, 0.038, and 0.005 (Fig. 7d). Overall, these findings suggest that both children and adults are at a heightened risk of developing cancer due to exposure to Cd, Cr, and Pb in water resources within the Gulf of Suez. Predicted cancer risk levels from the Monte Carlo simulation exceed the acceptable threshold (CR > 1.0E-04) in most water samples, emphasizing the potential for cancer development with sustained exposure to these metals. These results underscore the need to minimize metal contamination in water sources to mitigate carcinogenic health risks for adults and children.

The comparison between the calculated CR (Fig. 5) and predicted CR (Fig. 7) through dermal contact with heavy metals reveals a consistently high carcinogenic impact of the three metals on children and adults across most water samples collected from the Gulf of Suez. The close alignment and calibration of the calculated and predicted CR values underscores the effectiveness of the Monte Carlo simulation in predicting CR dermal values. This successful simulation application emphasizes its reliability in assessing the carcinogenic impact of heavy metals through dermal exposure, providing valuable insights for understanding and managing health risks associated with metal contamination in water sources.

### Statistical analysis and pollution source identification

#### Cluster analysis

**Fig. 8:**
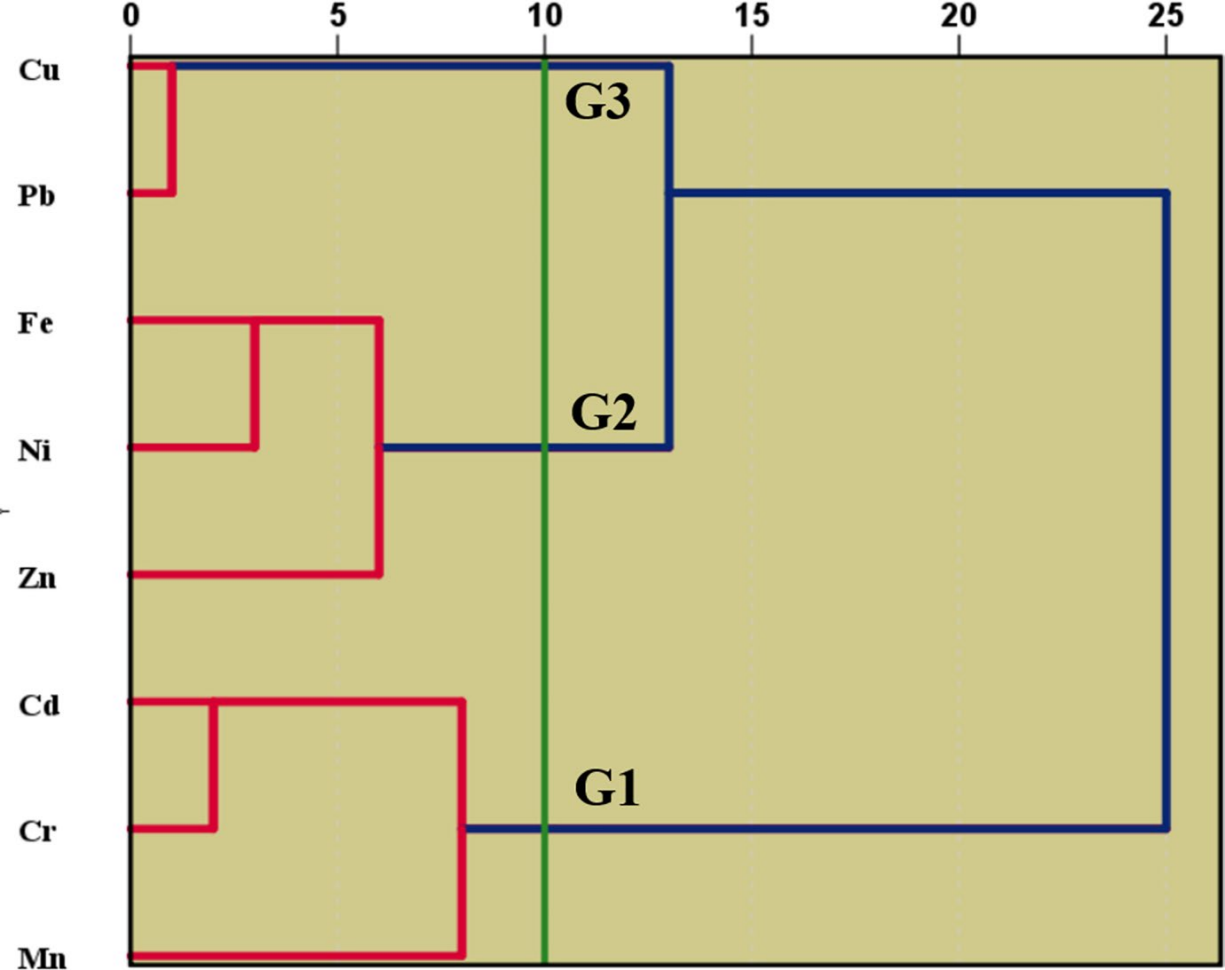
Cluster analysis (dendrogram) of the heavy metals in the collected samples.

The identification of three distinct groups in the analysis of coastal saltwater from the Red Sea (Fig. 8), achieved through a hierarchical cluster analysis employing the Wards linkage method and Euclidean distance, underscores the significance of using advanced analytical techniques for studying the chemical attributes of heavy metals in marine environments. The analysis of seawater samples from the Red Sea identified distinct chemical clusters, shedding light on potential contamination sources and guiding tailored management strategies. In Cluster 1 (Cadmium, Chromium, and Manganese), industrial discharges along the coastline are implicated, emphasizing the need for strict regulations, ongoing monitoring, and environmentally conscious industrial practices. Cluster 2 (Nickel, Zinc, and Iron) suggests a connection to petroleum-related activities, emphasizing the importance of thorough inspections, maintenance of offshore platforms, and robust response plans for potential oil spills. Cluster 3 (Copper and Lead) points to contamination sources in urban runoff from tourist areas and leaching from desalination station infrastructure. Effective management entails promoting responsible tourism, sustainable coastal development, and employing corrosion-resistant materials in desalination infrastructure. The presence of organic compounds, likely from anthropogenic activities, underscores the importance of identifying and controlling pollution sources and implementing advanced treatment methods, such as activated carbon filtration, before desalination. This site-specific approach, tailored to the characteristics of each cluster, aims to tackle heavy metal contamination challenges in the Red Sea proactively and efficiently.

#### Principal component analysis

**Fig. 9:**
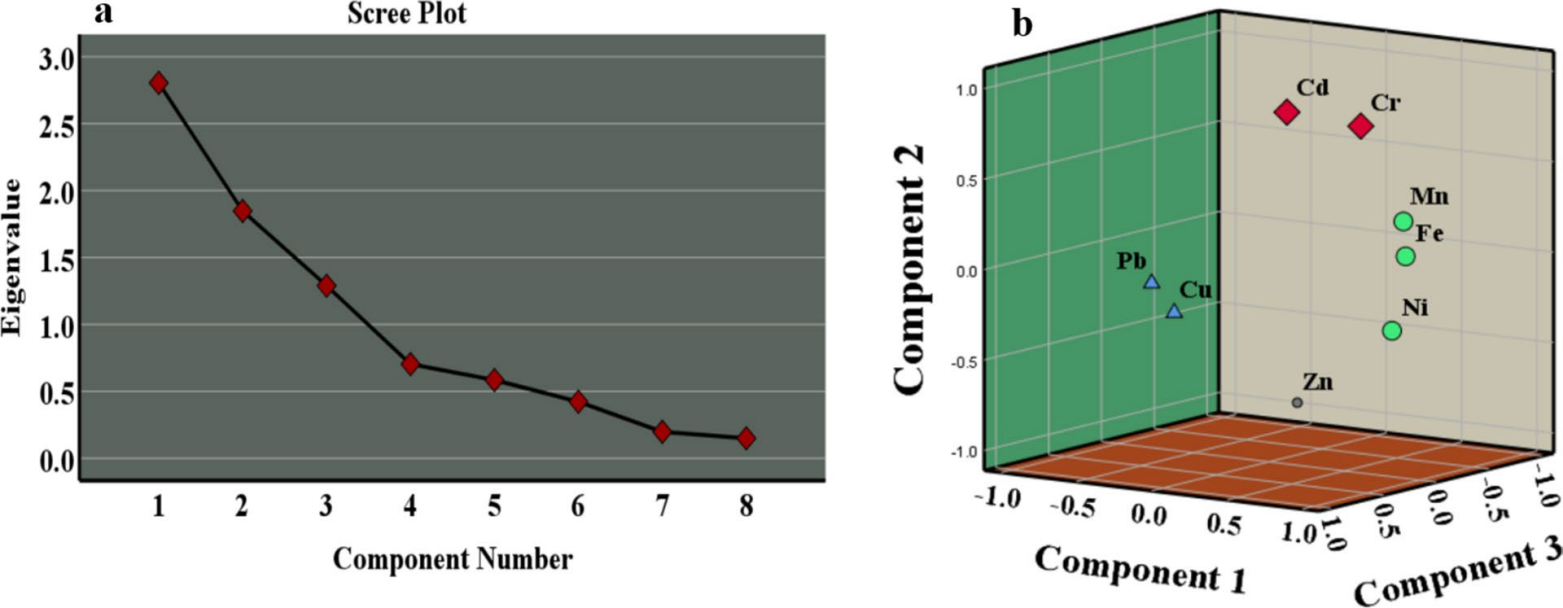
PCA extracted from (**a**) scree plot and its plotting on 3D diagram (**b**).

The principal component analysis (PCA) proved to be an invaluable tool in simplifying the intricacies of the water chemistry dataset, revealing underlying patterns and providing a nuanced understanding. The decision to utilize PCA was supported by a favorable Kaiser Meyer Olkin (KMO) value of 0.6, surpassing the acceptable threshold of 0.5. Bartlett's test of sphericity, yielding a significant result (0.02 < 0.05), further validated the appropriateness of the data for PCA. The scree plot (Fig. 9a) identified three principal components (PC1, PC2, and PC3), each with eigenvalues exceeding 1 (Fig. 9a,b). Collectively, these components elucidated 74.26% of the data's variability, with PC1 contributing 35.05%, PC2 23.09%, and PC3 16.11% to the cumulative explained variance. Examining variable loadings highlighted the robust associations between principal components and original variables. PC1 showcased a strong link with Fe (0.9), Ni (0.82), and Mn (0.64) (Table 3s). PC2 revealed a notable positive relationship with Cd (0.77) and Cr (0.73), coupled with a significant negative correlation with Zn (-0.79). PC3 demonstrated a substantial positive correlation with Cu (0.86) and Pb (0.9) (Table 3s). These findings underscore the pivotal variables influencing each principal component, offering valuable insights into the dominant factors shaping water chemistry patterns in the Red Sea. The cumulative explained variance emphasizes PCA's efficacy in capturing a substantial portion of the dataset's variability, complementing the insights derived from the hierarchical cluster analysis. The outcomes derived from the principal component analysis (PCA) seamlessly align with and enhance the insights garnered from the hierarchical cluster analysis, offering a holistic comprehension of heavy metal chemical attributes in the coastal saltwater of the Red Sea. The hierarchical cluster analysis unveiled three unique groups, each representing a specific chemical cluster with distinct heavy metal characteristics, and these clusters find further affirmation and elucidation through the results obtained from PCA. In the hierarchical cluster analysis, the identification of Cluster 1 (Cadmium, Chromium, and Manganese) linked to industrial discharges along the coastline receives robust support from PCA. The pronounced association between PC1 and variables such as Fe, Ni, and Mn in PCA underlines the influence of industrial activities in this cluster. Likewise, Cluster 2 (Nickel, Zinc, and Iron) in the hierarchical cluster analysis, indicating ties to petroleum-related activities, aligns seamlessly with PCA. The positive correlation of PC2 with variables like Cd and Cr and the negative correlation with Zn resonates with the indications of petroleum-related activities. Cluster 3 (Copper and Lead) receives confirmation through PCA in the hierarchical cluster analysis, hinting at contamination from urban runoff and desalination station infrastructure. The robust positive correlation between PC3 and variables Cu and Pb solidifies the association with these specific heavy metals. The overall consistency between hierarchical cluster analysis and PCA underscores the reliability of the findings. Both methodologies converge on identifying unique chemical clusters, offering valuable insights into potential contamination sources and guiding targeted management strategies to address the challenges of heavy metal contamination in the Red Sea.

### Reasearch work limitation and future work

This study underscores the importance of adopting a holistic approach to combat heavy metal pollution, considering the broader environmental context and advocating for proactive measures. However, there are certain limitations in the current research that could be addressed in future studies. One key aspect for further exploration involves measuring heavy metal and PAHs in the sediments and PTEs concentrations in water samples after the desalination process. This future work could offer more reliable insights into the health risks associated with drinking water. To enhance our understanding of heavy metal dynamics, future research should investigate how concentrations vary over time post-desalination, covering different seasons and years. This comprehensive approach could provide valuable insights into the long-term effects of metal pollution. Additionally, exploring various water treatment technologies and their effectiveness in reducing metal levels would contribute to the development of more efficient mitigation strategies. Understanding the social impacts of metal contamination on communities and industries is crucial for formulating holistic management strategies. It is essential to consider not only the environmental implications but also the broader socio-economic consequences. Lastly, further exploration into the feasibility and consequences of implementing suggested measures, such as desalination stations and salt extraction companies, would offer practical insights for effective environmental management in the Gulf of Suez.

## Conclusion

This in-depth study delves into the potential environmental and health hazards linked to PTEs and polycyclic aromatic hydrocarbons (PAHs) along the western coast of the Gulf of Suez. The investigation, centering on 16 PAHs, highlights concerning concentrations of pyrene (Pyr), particularly in the Suez area influenced by nearby refineries. Ratios confirming petrogenic sources point to the impact of industrial and shipping activities. Furthermore, heightened organic pollution in Suez Bay, exacerbated by oil rigs and refineries, is indicated by elevated levels of total organic carbon (TOC). The ecological risk assessment underscores significant risks, especially in Suez, emphasizing the urgent need for interventions to combat PAH contamination and protect aquatic ecosystems in the Red Sea. The identified clusters of industrial, petroleum-related, and urban runoff contamination are consistently validated by PCA. Notably, the Hazard Pollution Index (HPI) records a mean value of 537.8, exceeding permissible limits in 83.33% of samples, indicating significant contamination. The Metal Index (MI) ranges from 1.5 to 101.2, with 55.55% of samples categorized as severely affected. The Hazard Index (HI), calculated by summing hazard quotients (HQs) for each heavy metal and exposure route, ranges from 0.23 to 12.94 for adults and 0.89 to 49.39 for children through oral exposure. Through dermal exposure, HI values range from 0.02 to 0.81 for adults and 0.05 to 2.4 for children. Analysis reveals that HI oral values for adults and children exceeded safe levels (HI > 1) in 61.11% and 88.88% of water samples, respectively, indicating a high-risk category for non-carcinogenic impact. Conversely, HI values for adults indicated that 100% of water samples fell within the low-risk category for dermal contact. However, HI values for children showed 61.11% in the low-risk class and 38.88% in the high-risk category for dermal contact. The Monte Carlo simulation results provide valuable insights into the non-carcinogenic health risks associated with heavy metal exposure through oral and dermal pathways. For adults, the HQ oral values were generally below 1, indicating low non-carcinogenic risk for most metals except for Pb, which showed a higher risk. In contrast, children exhibited higher non-carcinogenic risks (HQ oral > 1) for Cd, Cr, and Pb, highlighting potential health concerns in this age group. Interestingly, the predicted HQ dermal values for adults and children were consistently below 1 for all eight metals, suggesting dermal exposure poses a lower non-carcinogenic risk. Addressing contamination before the desalination process is pivotal, as the identified pollutants not only impact desalination efficiency but also pose potential health risks if not mitigated. Immediate remediation efforts, stringent regulations, and enhanced monitoring are recommended, especially in high-risk areas like Suez. Implementing pre-desalination treatment methods, such as activated carbon filtration, is essential to ensure the quality of the produced water. Long-term monitoring, public awareness campaigns, and collaborative initiatives involving government, industries, and local communities are vital for sustained environmental health and the region's water sustainability. This study provides a roadmap for proactive measures, underlining the critical need to minimize contamination before embarking on the desalination process. The identified clusters and novel insights contribute to the development of targeted management strategies for effectively addressing heavy metal and PAH contamination challenges in the Red Sea. The future work can include monitoring the concentration of PTEs and PAHs in the sediments.

## Supplementary Information

Supplementary Tables.

## Data Availability

The datasets utilized and/or analyzed during the current study are available upon request from the corresponding author.
