# Supplementary material for: Environmental and health risk assessment of polycyclic aromatic hydrocarbons and toxic elements in the red sea using Monte Carlo simulation
Source: Sci Rep. 2025 Feb 3;15:4122. doi: 10.1038/s41598-024-71547-4 (PMC11790872; doi:10.1038/s41598-024-71547-4)
Supplement: Supplementary file 1 — Supplementary Tables. [file 41598_2024_71547_MOESM1_ESM.docx]

**Environmental and Health Risk Assessment of Polycyclic Aromatic Hydrocarbons and Toxic Elements in the Red Sea Using Monte Carlo Simulation**

**Alshaima Sayed F^a,b^; Mohamed Hamdy Eid٭^c, d^; Ahmed M. El-Sherbeeny^e^; Gouda Ismail Abdel-Gawad^d^; Essam A. Mohamed^a^; Mostafa R. Abukhadra^b,d^**

**Table 1s.** The parameters used for the calculation of HQ, HI, and CR.

| HM | Cr | Cd | Cu | Fe | Ni | Pb | Mn | Zn | Ref. |
| --- | --- | --- | --- | --- | --- | --- | --- | --- | --- |
| RfD Oral(mg/kg/day) | 0.003 | 0.0005 | 0.04 | 0.7 | 0.02 | 0.0014 | 0.024 | 0.3 | ^44^ |
| ABS | 0.025 | 0.05 | 0.3 | 0.2 | 0.04 | 0.3 | 0.04 | 0.2 | ^45^ |
| Rfd Dermal (mg/kg/day) | 0.000075 | 0.000025 | 0.012 | 0.14 | 0.0008 | 0.00042 | 0.00096 | 0.06 | ^46^ |
| CSF oral mg/kg/day | 0.5 | 6.1 |  |  |  | 0.5 |  |  | ^47^ |
| CSF dermal | 500 | 6100 |  |  |  | 500 |  |  | ^47^ |
| Kp | 0.002 | 0.001 | 0.001 | 0.001 | 0.0002 | 0.0001 | 0.001 | 0.0006 | ^48^ |
| Si | 0.05 | 0.003 | 3 | 0.3 | 0.07 | 0.01 | 0.05 | 1 | ^49^ |
| ET Adult (h/day) | 0.58 | | | | | | | | ^50^ |
| ET Child (h/day) | 1 | | | | | | | | ^50^ |
| SA Adult (cm^2^) | 18000 | | | | | | | | ^45^ |
| SA Child (cm^2^) | 6600 | | | | | | | | ^45^ |
| CF (L/cm^3^) | 0.001 | | | | | | | | ^50^ |
| IR Adult (L/day) | 2.2 | | | | | | | | ^46^ |
| IR Child (L/day) | 1.8 | | | | | | | | ^46^ |
| EF (day/year) | 350 | | | | | | | | ^41^ |
| ED Adult (year) | 70 | | | | | | | | ^45^ |
| ED Child (year) | 6 | | | | | | | | ^45^ |
| BW Adult (kg) | 70 | | | | | | | | ^51^ |
| BW Child (kg) | 15 | | | | | | | | ^51^ |
| AT Adult (day) | 25550 | | | | | | | | ^52^ |
| AT Child (day) | 2190 | | | | | | | | ^52^ |

Table 2s. The risk classification of the polycyclic aromatic hydrocarbons

| Parameter | Risk grade | RQ_NCs_ | RQM_PCs_ |
| --- | --- | --- | --- |
| Individual PAHs | Very low risk | ~ 0 |  |
|  | Medium risk | ≥ 1 | < 1 |
|  | High risk |  | ≥ 1 |
| Total PAHs | Very low risk | ~ 0 |  |
|  | Low risk | < 800 | ~ 0 |
|  | Medium risk 1 | ≥ 800 | ~ 0 |
|  | Medium risk 2 | < 800 | ≥ 1 |
|  | High risk | ≥ 800 | ≥ 1 |

**Table 3s** The tree component extracted from PCA and its correlation with heavy metals

| Parameters | PC1 | PC2 | PC3 |
| --- | --- | --- | --- |
| Fe | 0.9 | 0.114 | -0.01 |
| Ni | 0.82 | -0.303 | 0.005 |
| Mn | 0.644 | 0.231 | -0.354 |
| Zn | 0.078 | -0.799 | -0.157 |
| Cd | -0.063 | 0.778 | -0.261 |
| Cr | 0.391 | 0.739 | -0.312 |
| Pb | -0.146 | -0.008 | 0.903 |
| Cu | -0.02 | -0.163 | 0.869 |
| Eigenvalues | 2.805 | 1.848 | 1.289 |
| % of Variance | 26.016 | 24.679 | 23.575 |
| Cumulative % | 26.016 | 50.694 | 74.269 |
